# Taxane-Induced Peripheral Neurotoxicity

**DOI:** 10.3390/toxics3020152

**Published:** 2015-04-28

**Authors:** Roser Velasco, Jordi Bruna

**Affiliations:** 1 Unit of Neuro-Oncology, Hospital Universitari de Bellvitge-ICO Duran i Reynals, 08907 L´Hospitalet de Llobregat, Barcelona, Spain; E-Mail: rvelascof@bellvitgehospital.cat; 2 Institute of Neurosciences, Department of Cell Biology, Physiology and Immunology, Universitat Autònoma de Barcelona, and Centro de Investigación Biomèdica en Red sobre Enfermedades Neurodegenerativas (CIBERNED), 08193 Bellaterra, Spain

**Keywords:** taxane, paclitaxel, docetaxel, nab-paclitaxel, cabazitaxel, chemotherapy-induced peripheral neuropathy, neurotoxicity, peripheral neuropathy, taxane-induced peripheral neuropathy

## Abstract

Taxane-derived agents are chemotherapy drugs widely employed in cancer treatment. Among them, paclitaxel and docetaxel are most commonly administered, but newer formulations are being investigated. Taxane antineoplastic activity is mainly based on the ability of the drugs to promote microtubule assembly, leading to mitotic arrest and apoptosis in cancer cells. Peripheral neurotoxicity is the major non-hematological adverse effect of taxane, often manifested as painful neuropathy experienced during treatment, and it is sometimes irreversible. Unfortunately, taxane-induced neurotoxicity is an uncertainty prior to the initiation of treatment. The present review aims to dissect current knowledge on real incidence, underlying pathophysiology, clinical features and predisposing factors related with the development of taxane-induced neuropathy.

## 1. Introduction

Taxane is a family of antineoplastic agents discovered in the 1960s as a result of a National Cancer Institute screening program in which extracts from thousands of plants were tested for anticancer activity [1]. Taxanes have broad-spectrum activity in solid tumor malignancies, and are currently used as first-line treatment for breast, stomach, lung, head and neck, prostate and gynecological malignancies [2], which represent highly prevalent tumors in the general population. Currently, taxane-based chemotherapy schedules are a first-line treatment in both early-stage and metastatic breast cancer [3], the most common malignant tumor in women, occurring in one out of every eight women in her lifetime. Furthermore, recent research from experimental and clinical trials has demonstrated potential therapeutic effects of some type of taxane in several non-cancer diseases [4].

**Figure 1:**
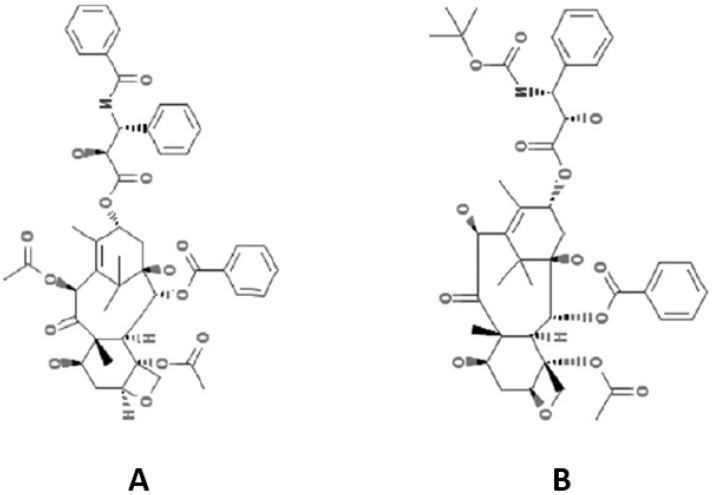
Chemical structure of paclitaxel (**A**) and docetaxel (**B**) (*Source*: http://pubchem.ncbi.nlm.nih.gov).

Paclitaxel and docetaxel were the initial prototypic taxane agents introduced into clinical practice, and they are two of the antineoplastic drugs currently in widest use. Paclitaxel was originally extracted from the bark of the yew tree (*Taxus brevifolia*) in the western region of the United States [1]. The most commonly used commercial preparation of paclitaxel is Taxol^®^ (Bristol-Myers Squibb; New York, NY, USA). This is a semisynthetic agent derived from the precursor 10-deacetylbaccatin III produced from the needles of European yew tree *Taxus baccata.* Docetaxel is a semisynthetic paclitaxel derivate commercialized under the trade name Taxotere^®^ (Sanofi-Aventis; Paris, France). Both paclitaxel and docetaxel consist of a complex taxane ring linked to an ester at the C-13 position [1] (Figure 1). Both share hydrophobic properties, requiring solvents for their administration: Cremophor^®^ EL (polyethoxylated castor oil, CrEL) for paclitaxel and polysorbate-80 for docetaxel. These solvents have been related with frequently observed allergic reactions [1,5]. Extensive research in new analogs and new formulations with better therapeutic and toxicity profiles, and higher solubility, is underway. The second-generation of taxane includes nanoparticle albumin-bound paclitaxel (nab-paclitaxel, ABI-007, Abraxane^®^) and cabazitaxel (Jevtana^®^), a semi-synthetic derivative of docetaxel, which have recently been incorporated in the antineoplastic armamentarium [5,6]. Liposomal paclitaxel formulations (*i.e*., Genexol^®^-PM) and other drug delivery systems are being investigated [7].

Paclitaxel and docetaxel are both associated with two main types of toxicity: peripheral neurotoxicity (NTX) and myelosuppression [1]. Whilst hematological toxicity can become lesser with the concurrent use of granulocyte colony-stimulating factors during chemotherapy, the occurrence of peripheral nerve toxicity represents one of the Achilles’ heels of taxane treatment. In one study including adjuvant or neoadjuvant breast cancer patients, dose-modification of taxane planned schedule was performed due to peripheral NTX in 17% of patients [8].

Taxane-induced peripheral neuropathy (TIPN) is problematic for several reasons. First, it is a growing issue due to improved long-term cancer survival. More cancer patients live longer or are cured due to the greater effectiveness of new drugs and therapeutic regimes. Second, receiving a complete taxane regimen is probably a critical factor in determining the outcome of a given cancer patient. TIPN is a dose-limiting side effect that must be considered when treating cancer patients with these agents, decreasing total cumulative dose administered [9], which may impair cancer outcome. Third, TIPN negatively impacts on routine activities, functions, and behaviors in the domestic, work, and social/leisure lives of cancer patients, adversely compromising the quality of the survivorship [10]. Finally, the development of TIPN is associated with an increase in the overall cost of cancer care for healthcare systems [11]. The present review is an attempt to dissect current knowledge on incidence, underlying pathophysiology, clinical features and predisposing factors related with the development of TIPN.

## 2. Epidemiology of TIPN

The incidence of all grades of TIPN among those patients treated with paclitaxel is high, ranging from 57%–83% overall and with severity in 2%–33% of patients [12]. Likewise, incidences reported with docetaxel are also highly variable: 11%–64% and 3%–14% overall and severe, respectively [13,14,15]. Although paclitaxel is classically considered more neurotoxic than docetaxel [8,16,17], one phase III clinical trial comparing paclitaxel 175 mg/m^2^ *vs*. docetaxel 100 mg/m^2^ every 21 days until progression or toxicity, reported a greater overall incidence of neuropathy in the docetaxel group (64.4% *vs*. 59%) and more frequent although, not significantly so, severe neuropathy (7.4% *vs*. 4.1%, *p* = 0.08) [14]. The substantial heterogeneity in reported rates when reviewing the literature can be partially explained by the differences in taxane schedules and the manner of NTX assessment [18]. The scales commonly used to evaluate TIPN employ a combination of subjective and objective items that are not always unambiguously described leading to variable interpretation. The limitations of the National Cancer Institute common toxicity criteria (NCI.CTC), including inconsistencies and broad generalization, explain much of the existing epidemiological confusion. Recently, one homogeneous study including more than 4500 breast cancer patients treated with taxane in the adjuvant setting reported an incidence of grades 2–4 NCI.CTC neuropathy according to as follows: 22% in paclitaxel weekly; 17.4% in paclitaxel every three weeks; 14.7% in docetaxel every three weeks; and 13.4% in docetaxel weekly-arm [19]. Of note, reported incidences emerge more clearly when detailed neurological examination is performed and neurological scales, such as the Total Neuropathy Score (TNS), are included in the TIPN assessment [20,21]. Pace *et al.* reported an incidence of 71% and 96% at 12 and 24 weeks of weekly-paclitaxel, respectively, assessed with neurologic and neurophysiological evaluation [22]. Published neuropathy incidence and severity also seem greater when patient-reported outcome measures are considered in TIPN assessment [23].

Regarding the second generation of taxane, phase III clinical trials comparing standard paclitaxel with nab-paclitaxel in breast cancer showed higher incidence of grade 3 neuropathy in nab-paclitaxel than in the standard treatment (10% *vs*. 2%) [24]. Conversely, lung cancer patients showed opposite ratios, 3% and 12% for nab-paclitaxel and standard treatment, respectively [25], in patients. On the other hand, an overall incidence of TIPN of 7.2%–14%, and a low-rate (1%) of severe neuropathy with cabazitaxel in the treatment of prostate cancer, have been consistently reported in clinical trials [26,27]. Finally, NTX rates up to 35% with liposomal paclitaxel formulation have been reported [28].

## 3. Pathophysiology of TIPN

Taxane belongs to a group of chemotherapy agents defined as “microtubule stabilizing agents” [29]. Taxane targets the cytoskeleton and spindle apparatus of tumor cells by binding to the microtubules, which are fibrillary polymers of α- and β-tubulin monomers. Taxane promotes microtubule assembly by stabilizing the polymerization of β-tubulin into bundles. This action disrupts mitotic spindles and arrests the cell cycle in the G0/G1 and G2/M phases leading to apoptosis in dividing cells [4,30,31]. However, neurons are quiescent cells. The exact pathophysiologic mechanism of TIPN is not well understood [32]. The inhibition of tubulin depolymerization and the consequent microtubule dysfunction seems the most widely accepted mechanism related with the taxane neurotoxic profile [32,33,34,35]. Intact microtubules are required for both anterograde and retrograde axonal transport, and neuronal survival and function depend on these transport processes. Increased axonal microtubule stability or polar reconfiguration [36] secondary to taxane might alter the retrograde axonal transport of growth factors, or other substances resulting in abnormal nerve physiology, and altered mitochondrial supply, leading to a loss of axonal integrity, or axonal degeneration in more severe cases. This phenomenon begins in the most vulnerable part of the nerve, the distal nerve endings of the longest nerves, where transport problems may manifest most quickly, and after this it spreads centrally, similar to other ‘dying back’-type peripheral neuropathies [33,34].

Paclitaxel does not cross the blood brain barrier, and its accumulation in the soma of sensory neurons of dorsal root ganglia (DRG) is presumably due to the relative deficiency in the blood–nerve barrier also observed in this region [34]. Microtubule impairment in the soma of the sensory neurons has been noted [34,37]. Furthermore, increased excitability of primary sensory neurons in DRG of animal models and changes in gene expression of some neuronal ion channels in DRG have recently been implicated in the development of paclitaxel-induced neuropathy [38].

Motor neurons are not as sensitive to taxane as sensory neurons are, despite their similar length. The mechanism by which taxane produces a preferential impairment of sensory neurons remains unclear [32]. Special vulnerability compared with motor neurons in the anterior horn of spinal cord was suggested [39]. Recent studies showed that mitochondria in sensory axons are more susceptible to the toxicity of paclitaxel compared to mitochondria in motor axons, providing a potential hypothesis for this fiber selectivity [40,41,42]. According to mitotoxicity hypothesis, the mitochondrial injury would lead to a chronic energy deficit in the axons causing abnormal spontaneous discharges and compartmental degeneration in somatosensory primary afferent neurons and that could be the common denominator in many distal symmetrical sensory neuropathies of different origin [42,43]. It has been attributed to the binding of paclitaxel to mitochondrial β-tubulin, which may produce Ca^2+^ release from mitochondria and dysregulated intracellular Ca^2+^ homeostasis [40]. However, why mitochondrial dysfunction in primary afferent sensory neurons would be predominant compared with other tissues with high energetic consumption is unresolved [32,42].

Another unanswered question is the pathogenic mechanisms underlying acute NTX related with taxane (see below), because this kind of NTX most likely is not primarily due to disorganization of microtubules and consequent disruption of axoplasmic flow in peripheral nerves [44]. The called “paclitaxel acute pain syndrome” (PAPS) has been related with a sensitization of nociceptors and their fibers by proinflammatory cytokines (IL-6, IL-8, IL1β, TNF-α) prior to the development of neuropathy [45,46,47].

On the other hand, morphological nerve changes observed in the only four patients treated with paclitaxel [48,49,50] and docetaxel [51] we are aware have been described in the literature in whom sural nerve biopsy was performed after finishing chemotherapy, showed severe nerve fiber loss, axonal atrophy, and secondary demyelination with a preferentially loss of large myelinated fibers [48]. Evidence of axonal regeneration was present in docetaxel [51] but not in paclitaxel [48,49,50]. Intriguingly, no aggregation of microtubules was observed on sural nerve biopsies from these patients [48,49,50].

Although it is well established that taxane affects large myelinated fibers (Aβ), the involvement of small myelinated (Aδ) and unmyelinated (C) sensory fibers is more controversial [31,44]. Experimental evidence shows that after paclitaxel infusion, pathological features occur in less than 5% of small DRG neurons and 30%–40% of large DRG neurons [52]. In patients, one clinical study including neurological examination and quantitative sensory testing showed predominant impairment of large myelinated fibers, intermediate impairment of Aδ fibers, and relative sparing of unmyelinated fibers [44]. Although there are numerous studies demonstrating loss of intraepidermal nerve fiber density (IEFND), a marker of small fiber impairment, in foot pad of animals treated with paclitaxel [53,54], evidence from patients is scarce. To our knowledge, only three pathological studies in patients evaluating small-fiber impairment by skin biopsy have been reported in the literature, with conflicting evidence. In one, including six patients prospectively assessed during treatment, the authors found an increase in all but one patient of IEFND during chemotherapy treatment [55]. A recently published cross-sectional study with 20 breast cancer women with established docetaxel-induced peripheral neuropathy reported a high proportion (15/20) of patients with loss of IEFND according to normative data [56], supporting small fiber involvement in the neuropathy due to docetaxel. Regarding paclitaxel, to our knowledge, available evidence is limited to one patient recently reported demonstrating a clear decrease in both Meissner’s corpuscule and IEFND densities as compared with healthy control subjects [57].

## 4. Clinical and Neurophysiological Pattern of TIPN

Patients undergoing taxane treatment may present two types of peripheral NTX: acute transient and subacute long-lasting TIPN. Paclitaxel and docetaxel are associated with an acute pain syndrome in up to 70% of patients [46], consisting of diffuse muscle aching, most often in the legs, hips, and lower back, although it can be widespread, regarded as myalgia or arthralgia that usually develops within one to three days of drug administration and usually resolves within a week [58,59,60]. Acute NTX is more frequently observed with short and high-dose infusions [46,61]. Despite its transient nature, it is highly distressing especially for paclitaxel-treated patients [60]. Interestingly, an association between the presence and severity of the PAPS and the further development of painful neuropathy has been reported. One detailed prospective study dissecting natural history of PAPS with 94 patients receiving paclitaxel reported that the initial intensity of pain experienced within the first week after the first dose of paclitaxel was predictive of the severity of shooting/burning pain symptoms of TIPN [46].

Long-lasting TIPN is characterized by symmetrical onset of sensory symptoms usually first in the tips of the toes and afterwards in the fingers, although simultaneous development in both the fingers and toes is not infrequent [21,44,62]. Sensory disturbance extends to soles and palms [44]. Facial involvement is less common, although anecdotally reported. In order of frequency, paclitaxel and docetaxel display a similar pattern of induced sensory symptoms including numbness (100%), tingling (80%), sensitivity to cold (60%) and neuropathic pain (50%) [44,60,62]. Loss of balance is also a complaint made by over 50% of patients [60]. Neurological examination consistently shows a loss of reflexes. Although initially only ankle reflexes are lost, global arreflexia is common. Of note, despite typical greater involvement of large diameter fibers, measurement of the vibration perception threshold seems not to be a reliable indicator of neuropathy and may be normal in many patients with TIPN [62]. Variable changes in pinprick and temperature sensibility can be found [44,62,63]. Romberg’s sign may be present in those patients most severely affected. Treatment with docetaxel has been associated with Lhermitte’s sign [62]. Conversely, motor impairment is unusual although muscle cramps, and distal muscle loss of strength may be observed. At high doses, proximal weakness can develop with severe muscle aches [64,65]. Nevertheless, when evident clinical motor signs occur, assessment by a neurologist is recommended to rule out other entities [65,66]. Symptoms associated with changes in autonomic function are infrequent but must be specifically interviewed in patients receiving taxane [62]. Autonomic cardiac and vascular dysfunction in the form of arrhythmias and orthostatic hypotension induced by paclitaxel have been reported [67], and demonstrated in comparative studies evaluating heart-rate recordings before and after taxane administration, with paclitaxel [68,69,70], but not with docetaxel [71].

Regarding time to onset of TIPN, paclitaxel induced neuropathy with one- and three-week schedules is reported to develop in 21 days (range 11–101) and 35 days (range 14–77), respectively [72]. Although a very early onset of grade 2 neuropathy after the first cycle of docetaxel can occur in up to one third of patients [15,44,62,72], one study comparing paclitaxel (175 mg/m^2^) and docetaxel (75 mg/m^2^) administered every three weeks found that the severity of TIPN peaked earlier (cycle 3 to cycle 7) in patients receiving paclitaxel compared with those receiving docetaxel (cycle 5–7 months) [73]. Further progression of neuropathy after finishing chemotherapy during 1–2 months (coasting effect) has been described with both agents [62].

Electrophysiological abnormalities when TIPN develops show an axonal peripheral neuropathy pattern, mainly involving the decrease of sensory nerve action potential or abolishment of sensory responses in sensory conduction studies. Reduction of compound motor action potential occurs at the highest cumulative doses, or may be normal in most cases, while sensory and motor nerve conduction velocity is usually spared. Needle electromyography may show variable amounts of abnormal spontaneous activity related with denervation depending on the severity of the neuropathy and degree of motor fiber involvement [62,65,66].

TIPN is considered to have an overall good long-term prognosis [66]. Despite appearing to be largely reversible, long-term studies with large follow-up reveal significant rates of persistent TIPN although usually well tolerated [74,75]. Overall, approximately half of patients with TIPN completely recover within the first year [72,73], to a lesser extent in those receiving docetaxel which seems to present more frequent severe residual TIPN compared with paclitaxel at 12 months [73]. However, most reported studies are retrospective in nature [66,75], include a highly variable frame of observation period of time [66,74,75] or variable manner of NTX gradation [66,73], which compromises comparison of reported frequencies among them.

## 5. Risk Factors for TIPN

Not all cancer patients receiving taxane will develop TIPN. Moreover, among those patients affected, even in the setting of uniform insult, there is considerable difference in the severity of NTX. Reasons underlying this variability are partially known, and there are no established, validated predictive biomarkers to determine which patients are at greater risk for TIPN. Furthermore, diagnosing and assessing symptoms related to TIPN in daily practice is complex and typically the physician goes after clinical syndrome. In particular, NTX in metastatic breast cancer patients receiving paclitaxel can be problematic because treatment is frequently administered until progression or toxicity, as a common approach. In general, chemotherapy-related and patient predisposing conditions should be considered before treatment with taxane in cancer patients.

### 5.1. Treatment Related Risk Factors

Total cumulative dose and dose intensity are considered the more important determinants of incidence and severity of NTX, as in other chemotherapy-induced peripheral neuropathies [18]. Onset doses for neuropathy of any grade range from 100–300 mg/m^2^ and 75–100 mg/m^2^ with paclitaxel and docetaxel, respectively [13]. In a randomized phase III study of metastatic breast cancer, the mean cumulative dose leading to onset of grade 2 peripheral NTX was 371 mg/m^2^ for docetaxel and 715 mg/m^2^ for paclitaxel [14]. Generally, severe TIPN occurs in patients receiving cumulative doses around 1000 mg/m^2^ for paclitaxel and 400 mg/m^2^ for docetaxel [29]. Traditionally, weekly paclitaxel schedules have been associated with higher NTX than tri-weekly schedules [76,77]. However, other studies comparing weekly and three-weekly paclitaxel schedules failed to find this association [72], including two recent meta-analyses of the available evidence from randomized controlled trials that observed less NTX with weekly paclitaxel compared with three-weekly schedules [78,79]. The relationship between weekly and every-three-week administration of docetaxel and NTX is uncertain, as studies have shown conflicting results [80,81]. The NTX induced by paclitaxel is also infusion-duration related, and most frequently occurs when paclitaxel is infused over 3 h instead of 24 h, suggesting that NTX is related to peak plasma concentration [82]. However, one study comparing weekly paclitaxel infused in 1 h or 3 h reported comparable frequency of NTX [83].

Notwithstanding, the administration of multiple neurotoxic agents concurrently is not uncommon in oncology practice. Co-administration of paclitaxel with cisplatin, which is a common schedule in a variety of cancers, showed that nearly the totality (95%) of patients developed peripheral NTX [65]. Concurrent treatment of taxane with other neurotoxic agents seems to induce a synergistic effect more than an additive one. Another study including 63 patients treated with docetaxel and cisplatin in which NTX was prospectively assessed in detail revealed that the neurotoxic effects of this combination were more severe than either cisplatin or docetaxel as a single agent at similar doses [62]. Another study comparing the safety of weekly cisplatin with or without paclitaxel demonstrated that the odds ratio for NTX was 8.3 for paclitaxel coadministration [84]. Regarding non-neurotoxic agents sometimes administered concurrently with taxane, bevacizumab has not been shown to modify the risk for NTX [85,86,87] despite experimental evidence supporting a potential NTX-inducing role [88].

### 5.2. Patient Predisposing Risk Factors

Demographic characteristics of patients receiving taxane have also been investigated to search for risk factors associated with TIPN development. Whereas it has been suggested that elderly people were more prone to developing TIPN [89,90], many studies have failed to observe such association [15,62,91] including the largest epidemiologic retrospective study published of breast, lung and ovarian cancer [92]. Regarding severity, more severe TIPN also seems not to be related with age of patients [91]. Nevertheless, one retrospective study evaluating duration of NTX induced by paclitaxel reported longer persistence of neuropathy in older patients (>60 years) [72]. In regard to race differences in TIPN, several paclitaxel studies show a higher associated risk of NTX in the African-American population [8], and found that non-Europeans were at significantly higher neuropathy risk than Europeans of similar genotype [93].

The putatively higher associated risk of TIPN in patients with pre-existing neuropathy is difficult to evaluate since the majority of clinical trials routinely exclude these patients. Whilst a statistically significant association was observed for early peripheral NTX induced by docetaxel and preexisting neuropathy [15], other studies failed to find this association [16,92]. More agreement exists regarding patients with the hereditary neuropathy Charcot-Marie-Tooth (CMT) being poorly tolerant of paclitaxel [16,94]. Furthermore, conflicting evidence regarding the potential higher-risk of TIPN in patients suffering from diabetes mellitus is also present in the literature [8]. Importantly, a lack of information concerning concurrent peripheral neuropathy secondary to diabetes is common and critical in these studies.

In addition, the role of tumor-related characteristics associated with TIPN has also been investigated. In one study including more than 1700 breast cancer patients, those who developed early docetaxel peripheral NTX more frequently had tumors less than 2 cm and node-negative disease [15]. However, most studies (all retrospective) including patients treated with paclitaxel and docetaxel [8,72] did not find any significant association of variables related with tumor type, extension and time to or duration of TIPN [72].

Finally, there is growing evidence that chemotherapy-related toxicity is an inheritable trait, and the genetic signature of a predisposition to TIPN is increasingly being investigated. Several studies have identified genetic polymorphisms associated with TIPN, most of them with inconclusive results [95,96,97,98,99,100], and only few accompanied with replication or validation studies [101,102,103]. Overall, polymorphisms in genes involved in the drug metabolism, distribution, and elimination properties of taxanes have been shown to be relevant for TIPN [102]. More recently, the role of genes involved in regulation of axon outgrowth [98] and genes associated with congenital neuropathies (Charcot-Marie-Tooth disease) [99] have been also reported in large prospective studies. However, the available data need to be interpreted with caution because of several limitations in the available studies, including the implementation of post-hoc analysis of oncology-based databases of different, not pre-planned size and inappropriate outcome measures for neurological impairment. For these reasons, it is not recommendable to adopt decisions on treatment on the basis of the current evidence on genomics.

## 6. Neuroprotection Strategies and Symptomatic Treatment for TIPN

Multiple strategies to prevent TIPN have been investigated, but no therapy has been definitely proven in randomized controlled trials to be beneficial for preventing the paclitaxel-associated acute pain syndrome or long-lasting TIPN. Among the strategies studied are acetyl-L-Carnitine, amifostine, amitriptyline, glutamate, glutathione, leukemia inhibitory factor, omega 3, corticosteroids, retinoid acid and Vitamin E [104,105,106,107,108].

Treatment of established TIPN aims to relieve disturbing symptoms such as neuropathic pain, numbness, and tingling. The types of drugs usually recommended by physicians include topical analgesics, antidepressants, and anticonvulsants, despite the lack of data from prospective placebo-controlled trials. Up to now, evidence concerning their effectiveness is scarce or negative [104,109]. Most robust evidence for treatment of TIPN emerges from a large randomized, double-blind, placebo-controlled phase III trial with duloxetine (60 mg/daily), which was shown to be modestly but significantly effective in diminishing TIPN-related pain [110]. In this trial, duloxetine, a selective serotonin and norepinephrin reuptake inhibitor used in depression and in diabetic neuropathy, was demonstrated to be more effective than placebo in chemotherapy-induced peripheral neuropathic pain, including 87 (40%) of patients treated with paclitaxel [110]. Of note, the exploratory subgroup analysis of the study suggested that duloxetine may work better for patients with painful oxaliplatin-induced, as opposed to painful TIPN. Importantly, attention was paid to the potential interaction of duloxetine in breast cancer patients with TIPN receiving tamoxifen, due to the fact that duloxetine inhibits CYP P450 2D6 enzyme, which is necessary to correct the conversion of tamoxifen to its active metabolite, endoxifen [110].

## 7. Conclusions

TIPN is of major concern for physicians and patients due to the negative consequences in terms of cancer outcome and quality of life. New taxane formulations are being developed to improve antineoplastic properties and minimize toxicities, but NTX remains an unsolved problem. There are limited data available describing risk factors of NTX, mainly epidemiologic and obtained from retrospective studies. Increased knowledge from pharmacogenomics studies is being investigated regarding inherited risk associated with TIPN development. However, knowledge of these risk markers is still far from being considered in clinical practice, and multiple considerations must be made before denying taxane treatment to candidate cancer patients based on the potential NTX savings. In this line, prospective neurological monitoring including subjective and objective clinical and neurophysiological data might be useful, as demonstrated in toxic neuropathies induced by oxaliplatin [111] and bortezomib [112]. The early identification of patients at risk of severe NTX by neurological monitoring could be useful to allow prompt reductions of the dose of taxane before irreversible or severe nerve damage occurs. Furthermore, knowledge of these predictive biomarkers may be critical to improving design of future clinical trials to test neuroprotective strategies against TIPN, which remains as a need.

## References

[1] Rowinsky E.K. (1997). The development and clinical utility of the taxane class of antimicrotubule chemotherapy agents. Annu. Rev. Med..

[2] Mekhail T.M., Markman M. (2002). Paclitaxel in cancer therapy. Expert Opin. Pharmacother..

[3] Bachegowda L.S., Makower D.F., Sparano J.A. (2014). Taxanes: Impact on breast cancer therapy. Anticancer Drugs.

[4] Zhang D., Yang R., Wang S., Dong Z. (2014). Paclitaxel: New uses for an old drug. Drug Des. Dev. Ther..

[5] Yared J.A., Tkaczuk K.H. (2012). Update on taxane development: New analogs and new formulations. Drug Des. Dev. Ther..

[6] Muggia F., Kudlowitz D. (2014). Novel taxanes. Anticancer Drugs.

[7] Zhang Z., Mei L., Feng S.S. (2013). Paclitaxel drug delivery systems. Expert Opin. Drug Deliv..

[8] Bhatnagar B., Gilmore S., Goloubeva O., Pelser C., Medeiros M., Chumsri S., Tkaczuk K., Edelman M., Bao T. (2014). Chemotherapy dose reduction due to chemotherapy induced peripheral neuropathy in breast cancer patients receiving chemotherapy in the neoadjuvant or adjuvant settings: A single-center experience. Springerplus.

[9] Speck R.M., Sammel M.D., Farrar J.T., Hennessy S., Mao J.J., Stineman M.G., DeMichele A. (2013). Impact of chemotherapy-induced peripheral neuropathy on treatment delivery in nonmetastatic breast cancer. J. Oncol. Pract..

[10] Ezendam N.P., Pijlman B., Bhugwandass C., Pruijt J.F., Mols F., Vos M.C., Pijnenborg J.M., van de Poll-Franse L.V. (2014). Chemotherapy-induced peripheral neuropathy and its impact on health-related quality of life among ovarian cancer survivors: Results from the population-based PROFILES registry. Gynecol. Oncol..

[11] Pike C.T., Birnbaum H.G., Muehlenbein C.E., Pohl G.M., Natale R.B. (2012). Healthcare costs and workloss burden of patients with chemotherapy-associated peripheral neuropathy in breast, ovarian, head and neck, and nonsmall cell lung cancer. Chemother. Res. Pract..

[12] Rivera E., Cianfrocca M. (2015). Overview of neuropathy associated with taxanes for the treatment of metastatic breast cancer. Cancer Chemother. Pharmacol..

[13] Stubblefield M.D., Burstein H.J., Burton A.W., Custodio C.M., Deng G.E., Ho M., Junck L., Morris G.S., Paice J.A., Tummala S. (2009). NCCN task force report: Management of neuropathy in cancer. J. Natl. Compr. Cancer Netw..

[14] Jones S.E., Erban J., Overmoyer B., Budd G.T., Hutchins L., Lower E., Laufman L., Sundaram S., Urba W.J., Pritchard K.I. (2005). Randomized phase III study of docetaxel compared with paclitaxel in metastatic breast cancer. J. Clin. Oncol..

[15] Eckhoff L., Knoop A.S., Jensen M.B., Ejlertsen B., Ewertz M. (2013). Risk of docetaxel-induced peripheral neuropathy among 1725 Danish patients with early stage breast cancer. Breast Cancer Res. Treat..

[16] Kudlowitz D., Muggia F. (2013). Defining risks of taxane neuropathy: Insights from randomized clinical trials. Clin. Cancer Res..

[17] Miltenburg N.C., Boogerd W. (2014). Chemotherapy-induced neuropathy: A comprehensive survey. Cancer Treat. Rev..

[18] Argyriou A.A., Bruna J., Marmiroli P., Cavaletti G. (2012). Chemotherapy-induced peripheral neurotoxicity (CIPN): An update. Crit. Rev. Oncol. Hematol..

[19] Schneider B.P., Zhao F., Wang M., Stearns V., Martino S., Jones V., Perez E.A., Saphner T., Wolff A.C., Sledge G.W. (2012). Neuropathy is not associated with clinical outcomes in patients receiving adjuvant taxane-containing therapy for operable breast cancer. J. Clin. Oncol..

[20] Cavaletti G., Frigeni B., Lanzani F., Piatti M., Rota S., Briani C., Zara G., Plasmati R., Pastorelli F., Caraceni A. (2007). The Total Neuropathy Score as an assessment tool for grading the course of chemotherapy-induced peripheral neurotoxicity: Comparison with the National Cancer Institute-Common Toxicity Scale. J. Peripher. Nerv. Syst..

[21] Park S.B., Lin C.S., Krishnan A.V., Friedlander M.L., Lewis C.R., Kiernan M.C. (2011). Early, progressive, and sustained dysfunction of sensory axons underlies paclitaxel-induced neuropathy. Muscle Nerve.

[22] Pace A., Nisticò C., Cuppone F., Bria E., Galiè E., Graziano G., Natoli G., Sperduti I., Jandolo B., Calabretta F. (2007). Peripheral neurotoxicity of weekly paclitaxel chemotherapy: A schedule or a dose issue?. Clin. Breast Cancer.

[23] Alberti P., Rossi E., Cornblath D.R., Merkies I.S., Postma T.J., Frigeni B., Bruna J., Velasco R., Argyriou A.A., Kalofonos H.P. (2014). Physician-assessed and patient-reported outcome measures in chemotherapy-induced sensory peripheral neurotoxicity: Two sides of the same coin. Ann. Oncol..

[24] Gradishar W.J., Tjulandin S., Davidson N., Shaw H., Desai N., Bhar P., Hawkins M., O’Shaughnessy J. (2005). Phase III trial of nanoparticle albumin-bound paclitaxel compared with polyethylated castor oil-based paclitaxel in women with breast cancer. J. Clin. Oncol..

[25] Hirsh V., Okamoto I., Hon J.K., Page R.D., Orsini J., Sakai H., Zhang H., Renschler M.F., Socinski M.A. (2014). Patient-reported neuropathy and taxane-associated symptoms in a phase 3 trial of nab-paclitaxel plus carboplatin *vs.* solvent-based paclitaxel plus carboplatin for advanced non-small-cell lung cancer. J. Thorac Oncol..

[26] Bahl A., Oudard S., Tombal B., Ozgüroglu M., Hansen S., Kocak I., Gravis G., Devin J., Shen L., de Bono J.S. (2013). Impact of cabazitaxel on 2-year survival and palliation of tumour-related pain in men with metastatic castration-resistant prostate cancer treated in the TROPIC trial. Ann. Oncol..

[27] De Bono J.S., Oudard S., Ozguroglu M., Hansen S., Machiels J.P., Kocak I., Gravis G., Bodrogi I., Mackenzie M.J., Shen L. (2010). Prednisone plus cabazitaxel or mitoxantrone for metastatic castration-resistant prostate cancer progressing after docetaxel treatment: A randomised open-label trial. Lancet.

[28] Ahn H.K., Jung M., Sym S.J., Shin D.B., Kang S.M., Kyung S.Y., Park J.W., Jeong S.H., Cho E.K. (2014). A phase II trial of Cremorphor EL-free paclitaxel (Genexol-PM) and gemcitabine in patients with advanced non-small cell lung cancer. Cancer Chemother. Pharmacol..

[29] Lee J.J., Swain S.M. (2006). Peripheral neuropathy induced by microtubule-stabilizing agents. J. Clin. Oncol..

[30] Rowinsky E.K., Donehower R.C. (1995). Paclitaxel (taxol). N. Engl. J. Med..

[31] Argyriou A.A., Koltzenburg M., Polychronopoulos P., Papapetropoulos S., Kalofonos H.P. (2008). Peripheral nerve damage associated with administration of taxanes in patients with cancer. Crit. Rev. Oncol. Hematol..

[32] Gornstein E., Schwarz T.L. (2014). The paradox of paclitaxel neurotoxicity: Mechanisms and unanswered questions. Neuropharmacology.

[33] Cavaletti G., Tredici G., Braga M., Tazzari S. (1995). Experimental peripheral neuropathy induced in adult rats by repeated intraperitoneal administration of taxol. Exp. Neurol..

[34] Cavaletti G., Cavalletti E., Montaguti P., Oggioni N., de Negri O., Tredici G. (1997). Effect on the peripheral nervous system of the short-term intravenous administration of paclitaxel in the rat. Neurotoxicology.

[35] Peters C.M., Jimenez-Andrade J.M., Kuskowski M.A., Ghilardi J.R., Mantyh P.W. (2007). An evolving cellular pathology occurs in dorsal root ganglia, peripheral nerve and spinal cord following intravenous administration of paclitaxel in the rat. Brain Res..

[36] Shemesh O.A., Spira M.E. (2010). Paclitaxel induces axonal microtubules polar reconfiguration and impaired organelle transport: Implications for the pathogenesis of paclitaxel-induced polyneuropathy. Acta Neuropathol..

[37] Scuteri A., Nicolini G., Miloso M., Bossi M., Cavaletti G., Windebank A.J., Tredici G. (2006). Paclitaxel toxicity in post-mitotic dorsal root ganglion (DRG) cells. Anticancer Res..

[38] Zhang H., Dougherty P.M. (2014). Enhanced excitability of primary sensory neurons and altered gene expression of neuronal ion channels in dorsal root ganglion in paclitaxel-induced peripheral neuropathy. Anesthesiology.

[39] Gregg R.W., Molepo J.M., Monpetit V.J., Mikael N.Z., Redmond D., Gadia M., Stewart D.J. (1992). Cisplatin neurotoxicity: The relationship between dosage, time, and platinum concentration in neurologic tissues, and morphologic evidence of toxicity. J. Clin. Oncol..

[40] Flatters S.J., Bennett G.J. (2006). Studies of peripheral sensory nerves in paclitaxel-induced painful peripheral neuropathy: Evidence for mitochondrial dysfunction. Pain.

[41] Xiao W.H., Zheng H., Zheng F.Y., Nuydens R., Meert T.F., Bennett G.J. (2011). Mitochondrial abnormality in sensory, but not motor, axons in paclitaxel-evoked painful peripheral neuropathy in the rat. Neuroscience.

[42] Bennett G.J., Doyle T., Salvemini D. (2014). Mitotoxicity in distal symmetrical sensory peripheral neuropathies. Nat. Rev. Neurol..

[43] Xiao W.H., Bennett G.J. (2012). Effects of mitochondrial poisons on the neuropathic pain produced by the chemotherapeutic agents, paclitaxel and oxaliplatin. Pain.

[44] Dougherty P.M., Cata J.P., Cordella J.V., Burton A., Weng H.R. (2004). Taxol-induced sensory disturbance is characterized by preferential impairment of myelinated fiber function in cancer patients. Pain.

[45] Loprinzi C.L., Maddocks-Christianson K., Wolf S.L., Rao R.D., Dyck P.J., Mantyh P., Dyck P.J. (2007). The Paclitaxel acute pain syndrome: Sensitization of nociceptors as the putative mechanism. Cancer J..

[46] Loprinzi C.L., Reeves B.N., Dakhil S.R., Sloan J.A., Wolf S.L., Burger K.N., Kamal A., Le-Lindqwister N.A., Soori G.S., Jaslowski A.J. (2011). Natural history of paclitaxel-associated acute pain syndrome: Prospective cohort study NCCTG N08C1. J. Clin. Oncol..

[47] Wang X.M., Lehky T.J., Brell J.M., Dorsey S.G. (2012). Discovering cytokines as targets for chemotherapy-induced painful peripheral neuropathy. Cytokine.

[48] Sahenk Z., Barohn R., New P., Mendell J.R. (1994). Taxol neuropathy. Electrodiagnostic and sural nerve biopsy findings. Arch. Neurol..

[49] Wiernik P.H., Schwartz E.L., Strauman J.J., Dutcher J.P., Lipton R.B., Paietta E. (1987). Phase I clinical and pharmacokinetic study of taxol. Cancer Res..

[50] Van den Bent M.J., van Raaij-van den Aarssen V.J., Verweij J., Doorn P.A., Sillevis Smitt P.A. (1997). Progression of paclitaxel-induced neuropathy following discontinuation of treatment. Muscle Nerve.

[51] Fazio R., Quattrini A., Bolognesi A., Bordogna G., Villa E., Previtali S., Canal N., Nemni R. (1999). Docetaxel neuropathy: A distal axonopathy. Acta Neuropathol..

[52] Mantyh P.W. (2006). Cancer pain and its impact on diagnosis, survival and quality of life. Nat. Rev. Neurosci..

[53] Boyette-Davis J., Xin W., Zhang H., Dougherty P.M. (2011). Intraepidermal nerve fiber loss corresponds to the development of taxol-induced hyperalgesia and can be prevented by treatment with minocycline. Pain.

[54] Ko M.H., Hu M.E., Hsieh Y.L., Lan C.T., Tseng T.J. (2014). Peptidergic intraepidermal nerve fibers in the skin contribute to the neuropathic pain in paclitaxel-induced peripheral neuropathy. Neuropeptides.

[55] Koskinen M.J., Kautio A.L., Haanpää M.L., Haapasalo H.K., Kellokumpu-Lehtinen P.L., Saarto T., Hietaharju A.J. (2011). Intraepidermal nerve fibre density in cancer patients receiving adjuvant chemotherapy. Anticancer Res..

[56] Krøigård T., Schrøder H.D., Qvortrup C., Eckhoff L., Pfeiffer P., Gaist D., Sindrup S.H. (2014). Characterization and diagnostic evaluation of chronic polyneuropathies induced by oxaliplatin and docetaxel comparing skin biopsy to quantitative sensory testing and nerve conduction studies. Eur. J. Neurol..

[57] Boyette-Davis J.A., Cata J.P., Driver L.C., Novy D.M., Bruel B.M., Mooring D.L., Wendelschafer-Crabb G., Kennedy W.R., Dougherty P.M. (2013). Persistent chemoneuropathy in patients receiving the plant alkaloids paclitaxel and vincristine. Cancer Chemother. Pharmacol..

[58] Rowinsky E.K., Eisenhauer E.A., Chaudhry V., Arbuck S.G., Donehower R.C. (1993). Clinical toxicities encountered with paclitaxel (Taxol). Semin. Oncol..

[59] Saibil S., Fitzgerald B., Freedman O.C., Amir E., Napolskikh J., Salvo N., Dranitsaris G., Clemons M. (2010). Incidence of taxane-induced pain and distress in patients receiving chemotherapy for early-stage breast cancer: A retrospective, outcomes-based survey. Curr. Oncol..

[60] Tofthagen C., McAllister R.D., Visovsky C. (2013). Peripheral neuropathy caused by paclitaxel and docetaxel: An evaluation and comparison of symptoms. J. Adv. Pract. Oncol..

[61] Moulder S.L., Holmes F.A., Tolcher A.W., Thall P., Broglio K., Valero V., Buzdar A.U., Arbuck S.G., Seidman A., Hortobagyi G.N. (2010). A randomized phase 2 trial comparing 3-h *vs.* 96-h infusion schedules of paclitaxel for the treatment of metastatic breast cancer. Cancer.

[62] Hilkens P.H., Verweij J., Vecht C.J., Stoter G., van den Bent M.J. (1997). Clinical characteristics of severe peripheral neuropathy induced by docetaxel (Taxotere). Ann. Oncol..

[63] Openshaw H., Beamon K., Synold T.W., Longmate J., Slatkin N.E., Doroshow J.H., Forman S., Margolin K., Morgan R., Shibata S. (2004). Neurophysiological study of peripheral neuropathy after high-dose Paclitaxel: Lack of neuroprotective effect of amifostine. Clin. Cancer Res..

[64] Freilich R.J., Balmaceda C., Seidman A.D., Rubin M., DeAngelis L.M. (1996). Motor neuropathy due to docetaxel and paclitaxel. Neurology.

[65] Chaudhry V., Rowinsky E.K., Sartorius S.E., Donehower R.C., Cornblath D.R. (1994). Peripheral neuropathy from taxol and cisplatin combination chemotherapy: Clinical and electrophysiological studies. Ann. Neurol..

[66] Osmani K., Vignes S., Aissi M., Wade F., Milani P., Lévy B.I., Kubis N. (2012). Taxane-induced peripheral neuropathy has good long-term prognosis: A 1- to 13-year evaluation. J. Neurol..

[67] Jerian S.M., Sarosy G.A., Link C.J., Fingert H.J., Reed E., Kohn E.C. (1993). Incapacitating autonomic neuropathy precipitated by taxol. Gynecol. Oncol..

[68] Ekholm E.M., Salminen E.K., Huikuri H.V., Jalonen J., Antila K.J., Salmi T.A., Rantanen V.T. (2000). Impairment of heart rate variability during paclitaxel therapy. Cancer.

[69] Ekholm E., Rantanen V., Antila K., Salminen E. (1997). Paclitaxel changes sympathetic control of blood pressure. Eur. J. Cancer.

[70] Barutcu I., Sezgin A.T., Gullu H., Esen A.M., Ozdemir R. (2004). Effect of paclitaxel administration on P wave duration and dispersion. Clin. Auton. Res..

[71] Ekholm E., Rantanen V., Syvänen K., Jalonen J., Antila K., Salminen E. (2002). Docetaxel does not impair cardiac autonomic function in breast cancer patients previously treated with anthracyclines. Anticancer Drugs.

[72] Tanabe Y., Hashimoto K., Shimizu C., Hirakawa A., Harano K., Yunokawa M., Yonemori K., Katsumata N., Tamura K., Ando M. (2013). Paclitaxel-induced peripheral neuropathy in patients receiving adjuvant chemotherapy for breast cancer. Int. J. Clin. Oncol..

[73] Shimozuma K., Ohashi Y., Takeuchi A., Aranishi T., Morita S., Kuroi K., Ohsumi S., Makino H., Katsumata N., Kuranami M. (2012). Taxane-induced peripheral neuropathy and health-related quality of life in postoperative breast cancer patients undergoing adjuvant chemotherapy: N-SAS BC 02, a randomized clinical trial. Support Care Cancer.

[74] Hershman D.L., Weimer L.H., Wang A., Kranwinkel G., Brafman L., Fuentes D., Awad D., Crew K.D. (2011). Association between patient reported outcomes and quantitative sensory tests for measuring long-term neurotoxicity in breast cancer survivors treated with adjuvant paclitaxel chemotherapy. Breast Cancer Res. Treat..

[75] Pignata S., de Placido S., Biamonte R., Scambia G., di Vagno G., Colucci G., Febbraro A., Marinaccio M., Lombardi A.V., Manzione L. (2006). Residual neurotoxicity in ovarian cancer patients in clinical remission after first-line chemotherapy with carboplatin and paclitaxel: The Multicenter Italian Trial in Ovarian cancer (MITO-4) retrospective study. BMC Cancer.

[76] Seidman A.D., Berry D., Cirrincione C., Harris L., Muss H., Marcom P.K., Gipson G., Burstein H., Lake D., Shapiro C.L. (2008). Randomized phase III trial of weekly compared with every-3-weeks paclitaxel for metastatic breast cancer, with trastuzumab for all HER-2 overexpressors and random assignment to trastuzumab or not in HER-2 nonoverexpressors: Final results of Cancer and Leukemia Group B protocol 9840. J. Clin. Oncol..

[77] Sparano J.A., Wang M., Martino S., Jones V., Perez E.A., Saphner T., Wolff A.C., Sledge G.W., Wood W.C., Davidson N.E. (2008). Weekly paclitaxel in the adjuvant treatment of breast cancer. N. Engl. J. Med..

[78] Huang T., Campbell T.C. (2012). Comparison of weekly *vs.* every 3 weeks paclitaxel in the treatment of advanced solid tumors: A meta-analysis. Cancer Treat. Rev..

[79] Mauri D., Kamposioras K., Tsali L., Bristianou M., Valachis A., Karathanasi I., Georgiou C., Polyzos N.P. (2010). Overall survival benefit for weekly *vs.* three-weekly taxanes regimens in advanced breast cancer: A meta-analysis. Cancer Treat. Rev..

[80] Di Maio M., Perrone F., Chiodini P., Gallo C., Camps C., Schuette W., Quoix E., Tsai C.M., Gridelli C. (2007). Individual patient data meta-analysis of docetaxel administered once every 3 weeks compared with once every week second-line treatment of advanced non-small-cell lung cancer. J. Clin. Oncol..

[81] Rivera E., Mejia J.A., Arun B.K., Adinin R.B., Walters R.S., Brewster A., Broglio K.R., Yin G., Esmaeli B., Hortobagyi G.N. (2008). Phase 3 study comparing the use of docetaxel on an every-3-week *vs.* weekly schedule in the treatment of metastatic breast cancer. Cancer.

[82] Smith R.E., Brown A.M., Mamounas E.P., Anderson S.J., Lembersky B.C., Atkins J.H., Shibata H.R., Baez L., DeFusco P.A., Davila E. (1999). Randomized trial of 3-h *vs.* 24-h infusion of high-dose paclitaxel in patients with metastatic or locally advanced breast cancer: National Surgical Adjuvant Breast and Bowel Project Protocol B-26. J. Clin. Oncol..

[83] Mielke S., Mross K., Gerds T.A., Schmidt A., Wäsch R., Berger D.P., Lange W., Behringer D. (2003). Comparative neurotoxicity of weekly non-break paclitaxel infusions over 1 *vs.* 3 h. Anticancer Drugs.

[84] De Jongh F.E., van Veen R.N., Veltman S.J., de Wit R., van der Burg M.E., van den Bent M.J., Planting A.S., Graveland W.J., Stoter G., Verweij J. (2003). Weekly high-dose cisplatin is a feasible treatment option: Analysis on prognostic factors for toxicity in 400 patients. Br. J. Cancer.

[85] Miles D.W., Chan A., Dirix L.Y., Cortés J., Pivot X., Tomczak P., Delozier T., Sohn J.H., Provencher L., Puglisi F. (2010). Phase III study of bevacizumab plus docetaxel compared with placebo plus docetaxel for the first-line treatment of human epidermal growth factor receptor 2-negative metastatic breast cancer. J. Clin. Oncol..

[86] Smith I.E., Pierga J.Y., Biganzoli L., Cortés-Funes H., Thomssen C., Pivot X., Fabi A., Xu B., Stroyakovskiy D., Franke F.A. (2011). First-line bevacizumab plus taxane-based chemotherapy for locally recurrent or metastatic breast cancer: Safety and efficacy in an open-label study in 2251 patients. Ann. Oncol..

[87] Robert N.J., Diéras V., Glaspy J., Brufsky A.M., Bondarenko I., Lipatov O.N., Perez E.A., Yardley D.A., Chan S.Y., Zhou X. (2011). RIBBON-1: Randomized, double-blind, placebo-controlled, phase III trial of chemotherapy with or without bevacizumab for first-line treatment of human epidermal growth factor receptor 2-negative, locally recurrent or metastatic breast cancer. J. Clin. Oncol..

[88] Verheyen A., Peeraer E., Nuydens R., Dhondt J., Poesen K., Pintelon I., Daniels A., Timmermans J.P., Meert T., Carmeliet P. (2012). Systemic anti-vascular endothelial growth factor therapies induce a painful sensory neuropathy. Brain.

[89] Akerley W., Herndon J.E., Egorin M.J., Lyss A.P., Kindler H.L., Savarese D.M., Sherman C.A., Rosen D.M., Hollis D., Ratain M.J. (2003). Weekly, high-dose paclitaxel in advanced lung carcinoma: A phase II study with pharmacokinetics by the Cancer and Leukemia Group B. Cancer.

[90] Chen Y.M., Perng R.P., Shih J.F., Tsai C.M., Whang-Peng J. (2005). Chemotherapy for non-small cell lung cancer in elderly patients. Chest.

[91] Argyriou A.A., Polychronopoulos P., Koutras A., Iconomou G., Gourzis P., Assimakopoulos K., Kalofonos H.P., Chroni E. (2006). Is advanced age associated with increased incidence and severity of chemotherapy-induced peripheral neuropathy?. Support Care Cancer.

[92] Nurgalieva Z., Xia R., Liu C.C., Burau K., Hardy D., Du X.L. (2010). Risk of chemotherapy-induced peripheral neuropathy in large population-based cohorts of elderly patients with breast, ovarian, and lung cancer. Am. J. Ther..

[93] Hertz D.L., Roy S., Motsinger-Reif A.A., Drobish A., Clark L.S., McLeod H.L., Carey L.A., Dees E.C. (2013). CYP2C8*3 increases risk of neuropathy in breast cancer patients treated with paclitaxel. Ann. Oncol..

[94] Weimer L.H., Podwall D. (2006). Medication-induced exacerbation of neuropathy in Charcot Marie Tooth disease. J. Neurol. Sci..

[95] Hertz D.L., Motsinger-Reif A.A., Drobish A., Winham S.J., McLeod H.L., Carey L.A., Dees E.C. (2012). CYP2C8*3 predicts benefit/risk profile in breast cancer patients receiving neoadjuvant paclitaxel. Breast Cancer Res. Treat..

[96] Bergmann T.K., Gréen H., Brasch-Andersen C., Mirza M.R., Herrstedt J., Hølund B., du Bois A., Damkier P., Vach W., Brosen K. (2011). Retrospective study of the impact of pharmacogenetic variants on paclitaxel toxicity and survival in patients with ovarian cancer. Eur. J. Clin. Pharmacol..

[97] Sissung T.M., Mross K., Steinberg S.M., Behringer D., Figg W.D., Sparreboom A., Mielke S. (2006). Association of ABCB1 genotypes with paclitaxel-mediated peripheral neuropathy and neutropenia. Eur. J. Cancer.

[98] Chhibber A., Mefford J., Stahl E.A., Pendergrass S.A., Baldwin R.M., Owzar K., Li M., Winer E.P., Hudis C.A., Zembutsu H. (2014). Polygenic inheritance of paclitaxel-induced sensory peripheral neuropathy driven by axon outgrowth gene sets in CALGB 40101 (Alliance). Pharmacogenomics J..

[99] Beutler A.S., Kulkarni A.A., Kanwar R., Klein C.J., Therneau T.M., Qin R., Banck M.S., Boora G.K., Ruddy K.J., Wu Y. (2014). Sequencing of Charcot-Marie-Tooth disease genes in a toxic polyneuropathy. Ann. Neurol..

[100] Gréen H., Söderkvist P., Rosenberg P., Mirghani R.A., Rymark P., Lundqvist E.A., Peterson C. (2009). Pharmacogenetic studies of Paclitaxel in the treatment of ovarian cancer. Basic Clin. Pharmacol. Toxicol..

[101] Baldwin R.M., Owzar K., Zembutsu H., Chhibber A., Kubo M., Jiang C., Watson D., Eclov R.J., Mefford J., McLeod H.L. (2012). A genome-wide association study identifies novel loci for paclitaxel-induced sensory peripheral neuropathy in CALGB 40101. Clin. Cancer Res..

[102] Abraham J.E., Guo Q., Dorling L., Tyrer J., Ingle S., Hardy R., Vallier A.L., Hiller L., Burns R., Jones L. (2014). Replication of genetic polymorphisms reported to be associated with taxane-related sensory neuropathy in patients with early breast cancer treated with Paclitaxel. Clin. Cancer Res..

[103] Apellániz-Ruiz M., Lee M.Y., Sánchez-Barroso L., Gutiérrez-Gutiérrez G., Calvo I., García-Estévez L., Sereno M., García-Donás J., Castelo B., Guerra E. (2015). Whole-exome sequencing reveals defective CYP3A4 variants predictive of paclitaxel dose-limiting neuropathy. Clin. Cancer Res..

[104] Hershman D.L., Lacchetti C., Dworkin R.H., Lavoie Smith E.M., Bleeker J., Cavaletti G., Chauhan C., Gavin P., Lavino A., Lustberg M.B. (2014). Prevention and management of chemotherapy-induced peripheral neuropathy in survivors of adult cancers: American Society of Clinical Oncology clinical practice guideline. J. Clin. Oncol..

[105] Pronk L.C., Hilkens P.H., van den Bent M.J., van Putten W.L., Stoter G., Verweij J. (1998). Corticosteroid co-medication does not reduce the incidence and severity of neurotoxicity induced by docetaxel. Anticancer Drugs.

[106] Leal A.D., Qin R., Atherton P.J., Haluska P., Behrens R.J., Tiber C.H., Watanaboonyakhet P., Weiss M., Adams P.T., Dockter T.J. (2014). Alliance for Clinical Trials in Oncology North Central Cancer Treatment Group/Alliance trial N08CA-the use of glutathione for prevention of paclitaxel/carboplatin-induced peripheral neuropathy: A phase 3 randomized, double-blind, placebo-controlled study. Cancer.

[107] Argyriou A.A., Chroni E., Koutras A., Iconomou G., Papapetropoulos S., Polychronopoulos P., Kalofonos H.P. (2006). Preventing paclitaxel-induced peripheral neuropathy: A phase II trial of vitamin E supplementation. J. Pain Symptom Manag..

[108] Kottschade L.A., Sloan J.A., Mazurczak M.A., Johnson D.B., Murphy B.P., Rowland K.M., Smith D.A., Berg A.R., Stella P.J., Loprinzi C.L. (2011). The use of vitamin E for the prevention of chemotherapy-induced peripheral neuropathy: Results of a randomized phase III clinical trial. Support Care Cancer.

[109] Pachman D.R., Watsonm J.C., Lustberg M.B., Wagner-Johnston N.D., Chan A., Broadfield L., Cheung Y.T., Steer C., Storey D.J., Chandwani K.D. (2014). Management options for established chemotherapy-induced peripheral neuropathy. Support Care Cancer.

[110] Smith E.M., Pang H., Cirrincione C., Fleishman S., Paskett E.D., Ahles T., Bressler L.R., Fadul C.E., Knox C., Le-Lindqwister N. (2013). Effect of duloxetine on pain, function, and quality of life among patients with chemotherapy-induced painful peripheral neuropathy: A randomized clinical trial. JAMA.

[111] Velasco R, Bruna J., Briani C., Argyriou A.A., Cavaletti G., Alberti P., Frigeni B., Cacciavillani M., Lonardi S., Cortinovis D. (2014). Early predictors of oxaliplatin-induced cumulative neuropathy in colorectal cancer patients. J. Neurol. Neurosurg. Psychiatry.

[112] Velasco R., Petit J., Clapés V., Verdú E., Navarro X., Bruna J. (2010). Neurological monitoring reduces the incidence of bortezomib-induced peripheral neuropathy in multiple myeloma patients. J. Peripher. Nerv. Syst..

